# Identifying metabolomic features that predict metastasis of melanoma from a primary site

**DOI:** 10.1186/2049-3002-2-S1-P67

**Published:** 2014-05-28

**Authors:** Xiaolei Shi, Zeping Hu, Elena Piskounova, Michalis Agathocleous, Sean Morrison, Ralph DeBerardinis

**Affiliations:** 1 Children’s Medical Center Reserach Institute,UT Southwestern Medical Center, Dallas, TX, USA; 2 Howard Hughes Medical Institute, Chevy Chase, MD, USA; 3 McDermott Center for Human Growth and Development, University of Texas Southwestern Medical Center, Dallas, TX, USA

## Background

About 90% of mortality associated with cancer is attributable to metastatic disease. Thus, our ability to treat cancer is largely dependent on the capacity to prevent metastasis. Metabolic reprogramming is recognized to support cellular transformation and tumor initiation; however, whether or how metabolism supports metastasis remains an open question. This study seeks to identify metabolic predictors of metastasis, with the rationale that understanding metabolic changes will lead to novel insights into metastasis and perhaps new therapies to treat metastatic disease.

## Materials and methods

A patient-derived xenograft model was used in which metastasis in mice strongly correlates with metastatic history of patient donors. Tumor cells were originally obtained from melanoma patients and passaged exclusively in highly immunocompromised mice [1]. In this study, 17 of such tumor lines were investigated including 6 lines with low metastatic potential, 9 high metastatic potential and 2 intermediate metastatic potential. Tumor cells were implanted subcutaneously into mice, yielding 62 tumors. 3-4 biopsies from each primary tumor were harvested, for a total of 185 samples. A mass spectrometry (MS)-based analytical platform was used to characterize >150 metabolites in each specimen. The relative abundance of metabolites that contribute most to the distinction of highly metastatic from inefficiently metastatic melanomas was examined using statistical tools.

## Results

Unsupervised hierarchical clustering revealed that in every case, a given tumor biopsy was most closely related to all other biopsies descended from the same parental tumor. In addition, as a frequent mutation in melanoma, BRAF-mutant tumors were easily distinguished from BRAF-wild type tumors by their metabolomic signatures. More importantly, several metabolic pathways were found altered significantly when metabolomics profiles between melanoma lines with high or low metastatic potentials were compared.

## Conclusions

The MS-based metabolomic analysis of patient-derived melanoma xenografts demonstrates that tumors contain individual metabolomic identities that can be tracked. It may be possible to identify metabolomic features from primary tumors that predict various aspects of tumor biology, including the propensity to metastasize. The altered metabolic pathways in highly metastatic melanomas are potential therapeutic targets.

## References

[B1] Quintana E Piskounova E Shackleton M Weinberg D Eskiocak U Fullen DR Johnson TM Morrison SJ Human melanoma metastasis in NSG mice correlates with clinical outcome in patients Sci Transl Med 2012 4 159ra149 23136044 10.1126/scitranslmed.3004599 PMC4501487

